# The mechanical properties of plastic evidence bags used for collection and storage of drug chemicals relevant to clandestine laboratory investigations

**DOI:** 10.1080/20961790.2017.1335459

**Published:** 2017-06-09

**Authors:** Harmonie Michelot, Barbara Stuart, Shanlin Fu, Ronald Shimmon, Tony Raymond, Tony Crandell, Claude Roux

**Affiliations:** a Centre for Forensic Science, University of Technology Sydney, Ultimo, NSW, Australia; b Strategic Forensic Support Branch, NSW Police Force, Parramatta, NSW, Australia; c Surry Hills Local Area Command, NSW Police Force, Surry Hills, NSW, Australia

**Keywords:** Forensic science, plastic evidence bags, drug seizures, tensile tests, tear tests

## Abstract

The effectiveness of three types of plastic bags used by the New South Wales Police Force for the storage of clandestine drug evidence has been investigated through a comparison of mechanical properties. The tensile and tear properties of “as received” low-density polyethylene (LDPE) and poly(vinyl chloride) (PVC) bags do not show major differences such that one type would be favoured over the other. However, the mechanical properties of the bags once exposed to a range of chemicals routinely collected as drug evidence have been shown to be influenced as a result of different chemical interactions. Although an interaction of reagents/solvents with an additive within the LDPE bags is proposed to influence the mechanical properties of the bags, the change in properties has been shown to be less severe than that observed for the PVC bag, where softening and damage of the bags results due to absorption of reagents.

## Introduction

Sealable plastic bags provide an effective means of transporting and storing a variety of types of police exhibits. This type of packaging has been widely adopted for evidence collection due to the ability to securely seal such bags, water resistance, transparency and the availability of a range of sizes [1,2]. Low-density polyethylene (LDPE) and poly(vinyl chloride) (PVC) are two polymers used in the production of evidence bags [3].

A form of seized materials for which plastic bags are commonly employed is illegally manufactured drugs and their associated precursor chemicals. Evidence plastic bags are particularly practical for the efficient and safe collection of drug evidence from clandestine laboratories. Despite the apparent inertness of plastic bags, anecdotal evidence suggests that after storage of some types of chemicals, the physical properties of the bags may be compromised [4]. It is acknowledged that liquids are usually stored in glass containers beforehand. However, cases have been reported where hazardous liquids had leaked and affected the plastic bag. In fact, the visual and chemical degradation of evidence bags was demonstrated in a previous study, and it was suggested to systematically ensure that seized chemicals were stored in proper containers before being put into the plastic bag [5].

This observation suggests that there is in addition of chemical interactions, some form of physical interaction between the stored chemical and evidence bag that potentially produces a deterioration in the properties of the bags.

The aim of this study was to investigate the physical properties of the plastic bags currently used by the NSW Police Force to determine their effectiveness for the collection and storage of drug and related chemicals typically seized by the police. Both LDPE and PVC bags have been employed in the field and bags provided by different manufacturers^1.^ have been in use for drug evidence. In some cases, the same polymer type has been used for manufacture, but the thickness of the plastic sheets employed to produce the bags vary, resulting in a perceived difference in durability for the collection of evidence (i.e. thicker films appear to practitioners to be less likely to tear when in use).

In order to determine the applicability of three types of evidence bags employed for the collection of drugs and their associated chemicals, a range of reagents and solvents frequently recovered from clandestine drug laboratories were stored in the bags under controlled environmental conditions. The physical properties of the plastic evidence bags have been investigated by measuring the mechanical properties via standard tensile and tear tests, to help the NSW Police force to get a better knowledge of evidence bags that are routinely used for the storage of drugs and related chemicals.

## Materials and methods

### Materials

Three types of plastic evidence bags were provided by the NSW Police Force: LDPE bags sourced from two different manufacturers (denoted LDPE 1 and LDPE 2) and PVC bags sourced from a third manufacturer (manufacturers names as well as polymers compositions are confidential). The three types of bags are sealable bags with adhesive closure.

Thirteen reagents regularly encountered in clandestine laboratories were examined at the request of the NSW Police based on the frequency of collection of such reagents: acetic acid (≥99.7%), acetone (≥99.9%), acetonitrile (≥99.9%), ethyl acetate (99.8%), formic acid (≥97%), hydrogen peroxide (30% in H_2_O), hydrochloric acid (37%), hydriodic acid (57% in H_2_O), hydrogen bromide (48% in H_2_O), methylamine (40% in H_2_O), methanol (99.8%), nitroethane (≥98%) and phosphoric acid (85% in H_2_O) (supplied by Sigma-Aldrich).

### Reagent storage experiments

The reagents were diluted with ultra-pure water based on the known reactivity of the reagent with the polymer of interest in order to potentially inhibit very fast reactions [6–8]. The dilutions are summarized in Table 1. Three replicates of each bag type was filled with 50 mL of chosen reagent (i.e. three replicates per bag type and per chemical used), sealed and stored at ambient temperature for three months in an upright position prior to testing [9]. Bags containing ultra-pure water were also established as controls for the experiment.

**Table 1.** Aqueous dilutions used for reagent storage tests [7,8].

Chemical	Bag material	Total dilution in water (%)
Acetic acid	LDPE	40
	PVC	3
Acetone	LDPE	50
	PVC	10
Acetonitrile	LDPE	50
	PVC	10
Ethyl acetate	LDPE	50
	PVC	10
Formic acid	LDPE	40
	PVC	40
Hydrogen peroxide	LDPE	50
	PVC	80
Hydrochloric acid	LDPE	30
	PVC	3
Hydroiodic acid	LDPE	30
	PVC	40
Hydrogen bromide	LDPE	5
	PVC	40
Methanol	LDPE	0
	PVC	0
Methylamine	LDPE	20
	PVC	20
Nitroethane	LDPE	60
	PVC	10
Phosphoric acid	LDPE	40
	PVC	70

### Mechanical testing

The tensile and tear properties of the aged bags were measured using an Instron 6022 Universal Testing Machine. Tensile testing was conducted according to ASTM D882 [10] and tear testing was carried out according to ASTM D1004 [11]. An initial strain rate of 100 mm/mm.min was used for all tests on standardized specimens of dimensions 50 mm × 20 mm. The thicknesses of the specimens were: LDPE 1 0.07 mm; LDPE 2 0.15 mm; PVC 0.12 mm. Ten replicates were performed on each of the three types of bags prior to reagent exposure to test data reproducibility. For the stored bags, results were given as the average of the three replicates, due to the reproducibility of the data.

## Results and discussion

### Mechanical properties of “as received” bags

The tensile force at break and percentage of elongation at break were determined for each “as received” bag material (Table 2). The tensile force enables the maximum force that the bag can sustain before fracture to be determined, while the elongation at break represents the capacity of the bag to be stretched without failure.

**Table 2.** Tensile testing results for original evidence bags (mean ± standard deviation, *n* = 10).

Bag property	LDPE 1	LDPE 2	PVC
Tensile force at break (N)	37 ± 7	70 ± 7	97 ± 8
Ultimate tensile strength (MPa)	26 ± 5	23 ± 2	40 ± 3
Elongation at break (%)	699 ± 0	700 ± 1	185 ± 22
Tear force at break (N)	6 ± 1	9 ± 1	9 ± 2

The highest force to break for the PVC bags is 96.59 N, with the thicker LDPE 2 bag requiring 70.25 N and the thinner LDPE 1 requiring only 36.58 N to break. The thickness of the LDPE bags also influences the strength of the bags. The Ultimate Tensile Strength (UTS) of each bag was also determined to take into account the different thicknesses of the “as received” bags. UTS values of 26, 23 and 40 MPa were determined for LDPE 1, LDPE 2 and PVC bags, respectively. Although the “as received” PVC bag demonstrates a higher strength compared to the LDPE bags, the value is not significantly greater. The percentage of elongation values indicates that prior to storage of chemicals the LDPE bags are able to extend by 698.88% before breaking, while the PVC elongates by 185.41% under the same conditions. Thus, the LDPE appear to be the more ductile material.

Tear testing of the bags enabled the tear force at break to be determined for each evidence bag and the values are listed in Table 2. The thicker LDPE 2 bag (0.15 mm) shows a somewhat better tear resistance with a tear force (9 ± 1) N compared to a value of (6 ± 1) N for the thinner LDPE 1 bag (0.07 mm). The PVC bag requires the same tear force (9 ± 2)N as that of the thicker LDPE 2 bag, indicating that there is no difference regarding the degree of their tear resistance.

### Mechanical properties of bags exposed to reagents and solvents

Tensile tests were also carried out on the three bag types after storage of chemicals for a period of three months. The obtained tensile force at break and percentage of elongation at break are provided in Table 3.

**Table 3.** Tensile testing results for three types of evidence bags exposed to reagents for three months (mean, *n* = 3).

	Tensile force at break (N)			Elongation at break (%)		
Chemical	LDPE 1	LDPE 2	PVC	LDPE 1	LDPE 2	PVC
Acetic acid	26.96	45.47	73.25	401.34	101.00	699.34
Acetone	21.18	55.44	96.65	406.74	97.28	651.94
Acetonitrile	24.23	52.97	80.38	410.08	98.00	652.28
Ethyl acetate	23.53	45.34	86.23	363.28	106.68	706.08
Formic acid	18.57	62.77	78.48	0.00	140.00	663.20
Hydriodic acid	22.00	38.02	94.11	470.54	123.80	324.88
Hydrochloric acid	21.18	49.47	76.44	243.34	79.88	526.00
Hydrogen bromide	23.34	41.59	81.77	316.80	168.00	696.40
Hydrogen peroxide	23.53	45.40	77.83	323.54	86.00	699.76
Methanol	18.70	47.82	58.57	331.54	54.48	699.28
Methylamine	21.43	51.77	91.63	259.68	146.14	592.68
Nitroethane	23.91	56.14	88.20	381.94	138.40	652.02
Phosphoric acid	21.24	32.87	76.84	161.20	79.88	296.68
Water	22.38	43.43	70.01	336.88	62.94	648.26

Note: Relative standard deviation (RSD) based on three measurements for each treatment and each bag was minimal and deemed not significant in the comparison of these results.

For both LDPE bag types a reduction in the tensile forces and percentage of elongation values is observed by comparison with the values reported for the unexposed bags (Table 2). It is noted that for the LDPE 2 bag the percentage of elongation values are reduced to a greater extent than for the LDPE 1 bag. The tear forces measured for the LDPE 1 bag exposed to chemicals do not show a significant change, while there is a small increase in the tear force for the LDPE 2 bag when exposed to the same chemicals.

The consistent reduction in tensile properties when exposed to each reagent or solvent is unlikely to be associated with polymer solubility as polyethylene is known to be unreactive to the chemicals used in this experiment [6]. However, there is evidence that the mechanical properties of commercial polyethylene bags can be influenced by exposure to liquids. For instance, the presence of water has been observed to modify the tensile properties of plastics (Table 3), and a previous study has reported a decrease in tensile force for a polyethylene bag exposed to an aqueous environment for a period of months [12]. It is possible that the changes observed in the current study are associated with an additive, a slip agent, used in the manufacture of the LDPE bags rather than the base polymer itself. Slip additives, commonly fatty acid amides, are routinely added to polyethylene for film formation in order to improve the frictional properties of the polymer (via migration to the film surface) and to avoid adhesion during manufacture. An attenuated total reflectance (ATR) infrared spectrum of the surface of the original LDPE bags indicated the presence of a slip agent on the surface of the bags used for this study [5]. Exposure to reagents and solvents for three months may be responsible for the migration of the slip agent from the polyethylene bags into the reagents or solvents stored in the bag. A previous study has indicated that amide slip additives are able to migrate from LDPE film into water [13]. It has also been demonstrated that such agents are able to leach from plastic containers when various solvents are stored in the containers [14]. Hence, the loss of the slip additive has the potential to contribute to the observed changes to the mechanical properties of the LDPE bags, as the resulting further migration of more slip additives to the surface can result in microvoids in the film. Such microstructural changes have been shown to have the ability to affect the tensile properties of polyethylene films [15].

The tensile force at break for the PVC bags was reduced after exposure to most solvents, but with several chemicals showed a minimal change to this property (Table 3). By comparison, exposure to the solvents had more influence on the percentage of elongation at break for the PVC bags, with elongation significantly increased after three months exposure to reagents and solvents in each case.

Overall, a small increase in tear force at break was observed when PVC was exposed to the chemicals (Table 4).

**Table 4.** Tear testing results for the three types of evidence bags exposed to chemicals for three months (mean, *n* = 3).

	Tear force at break (N)		
Chemicals	LDPE 1	LDPE 2	PVC
Acetic acid	7.44	9.98	9.28
Acetone	7.50	10.11	9.60
Acetonitrile	6.04	9.92	11.76
Ethyl acetate	7.50	10.75	13.04
Formic acid	6.23	11.19	9.28
Hydriodic acid	6.23	11.70	9.73
Hydrochloric acid	6.49	12.21	12.34
Hydrogen bromide	6.10	11.70	16.15
Hydrogen peroxide	7.31	14.24	13.10
Methanol	5.21	9.60	16.66
Methylamine	6.10	11.19	9.54
Nitroethane	6.36	13.99	14.31
Phosphoric acid	7.31	10.56	9.73
Water	7.57	10.87	12.91

Note: Relative standard deviation (RSD) based on three measurements for each treatment and each bag was minimal and deemed not significant in the comparison of these results.

A combined reduction in tensile force and increase in elongation can be associated with a further softening (or plasticization) of the PVC film. The plasticizer used in the supplied PVC bags is a phthalate ester, predominantly dioctyl phthalate (DOP) (identified by infrared spectroscopy), and the concentration of plasticizer provided by the supplier was stated to be in the range of 10%–30%. The introduction of plasticizer molecules into the polymer structure provides more free volume and so more chain flexibility: when the polymer molecule becomes more flexible, better elongation is observed but the tensile strength is lowered compared to the unplasticized polymer [16]. The additional plasticization process observed for the bags after exposure to the reagents and solvents indicates that the chemicals are migrating into the bags during the three-month exposure period. Although the PVC molecules themselves nor the plasticizer is significantly soluble in the chemicals investigated in this study [6,16], the ability of reagents and solvent molecules to migrate into the regions between the polymer molecules and occupied by the plasticizer is feasible. Earlier work has demonstrated that plasticized PVC can be further plasticized due to the ingress of additional solvent [17].

Further evidence of the migration of reagent and solvent molecules into the system is provided by the changes to the bags observed on exposure to certain solvents investigated in the current study. For the PVC bags, more significant changes were observed and a “wrinkled” appearance was noted after removal from particular reagent/solvent systems (methanol, nitroethane and ethyl acetate). The observed wrinkling may result when the surface of the film becomes higher in viscosity while the bottom of the film is still relatively fluid [18]. The process can result from rapid solvent loss from the surface, followed by later solvent loss from the lower layers. The subsequent solvent loss in the lower layers results in shrinkage, which pulls the surface layer into a wrinkled pattern. In any case, this confirms the reduced resistance of PVC bags after storage of chemicals, compared to LDPE bags.

## Conclusions

Prior to exposure to reagents or solvents both evidence bag types, LDPE and PVC, showed acceptable physical properties for use as storage bags. The PVC bags demonstrated a somewhat higher tensile strength than the LDPE bags studied. The polyethylene bags were more ductile that the PVC bags. All bags showed a similar tear resistance. When exposed to a range of chemicals for 3 months, the mechanical properties of the bags were differently affected. The LDPE bags showed a decreased tensile force and elongation at break when exposed to the reagents/solvents. The changes are attributed to the migration of a slip additive from the bag into the chemical in which it is immersed. The influence of chemical exposure on the mechanical properties of the PVC bags differed from that observed for LDPE: the elongation at break was notably increased while the tensile force decreased after exposure. The presence of plasticizer and the further softening of the material as a result of the migration of solvent molecules into the bags were responsible for the changed mechanical properties. Due to the nature of the interaction with a range of relevant chemicals, the LDPE bags appear to be the preferred option for the storage of drug related evidence. In addition to less visual changes to the polyethylene bags, the influence of chemical interaction on the physical properties was less prominent than in the case of the PVC bag. This study highlights the need to be aware of the potential reactions that may occur between stored chemicals and the additives present in otherwise chemically inert plastic bags. This can result in alteration of evidence bags and may be hazardous for end users. Nevertheless, it was already suggested with a previous study that chemicals should preferably be introduced in proper containers first, and these containers being placed in evidence bags afterwards. Further research may be conducted in this field, as there is potential to investigate tailored formulations for plastic bags that can minimize the potential for interaction with the chemicals most likely to be stored in drug evidence bags.

## Note

1. Names of manufacturers were kept confidential.
